# Machine learning-based diagnostic prediction of IgA nephropathy: model development and validation study

**DOI:** 10.1038/s41598-024-63339-7

**Published:** 2024-05-30

**Authors:** Ryunosuke Noda, Daisuke Ichikawa, Yugo Shibagaki

**Affiliations:** Division of Nephrology and Hypertension, Department of Internal Medicine, St. Marianna University School of Medicine, 2-16-1 Sugao, Miyamae-Ku, Kawasaki, Kanagawa 216-8511 Japan

**Keywords:** IgA nephropathy, Kidney biopsy, Artificial intelligence, Machine learning, Glomerulonephritis, Nephrology, Kidney, Kidney diseases, Diagnostic markers, Predictive markers, Diagnosis

## Abstract

IgA nephropathy progresses to kidney failure, making early detection important. However, definitive diagnosis depends on invasive kidney biopsy. This study aimed to develop non-invasive prediction models for IgA nephropathy using machine learning. We collected retrospective data on demographic characteristics, blood tests, and urine tests of the patients who underwent kidney biopsy. The dataset was divided into derivation and validation cohorts, with temporal validation. We employed five machine learning models—eXtreme Gradient Boosting (XGBoost), LightGBM, Random Forest, Artificial Neural Networks, and 1 Dimentional-Convolutional Neural Network (1D-CNN)—and logistic regression, evaluating performance via the area under the receiver operating characteristic curve (AUROC) and explored variable importance through SHapley Additive exPlanations method. The study included 1268 participants, with 353 (28%) diagnosed with IgA nephropathy. In the derivation cohort, LightGBM achieved the highest AUROC of 0.913 (95% CI 0.906–0.919), significantly higher than logistic regression, Artificial Neural Network, and 1D-CNN, not significantly different from XGBoost and Random Forest. In the validation cohort, XGBoost demonstrated the highest AUROC of 0.894 (95% CI 0.850–0.935), maintaining its robust performance. Key predictors identified were age, serum albumin, IgA/C3, and urine red blood cells, aligning with existing clinical insights. Machine learning can be a valuable non-invasive tool for IgA nephropathy.

## Introduction

IgA nephropathy (IgAN) is the most common primary glomerulonephritis worldwide, leading to end-stage kidney failure in 30–40% of patients within two decades of diagnosis^1^. For favorable outcomes in IgAN patients, early detection and timely treatment are essential. IgAN presents variable clinical courses, characterized by various degrees of hematuria and/or proteinuria, complicating diagnosis with general laboratory tests^2,3^. Definitive diagnosis requires kidney biopsy, which, however, has several contraindications and entails risks like significant bleeding^4^, which in severe cases, may require interventions such as transfusion, arterial embolization, or surgery, representing a crucial clinical challenge^5^.

The potential for predicting the diagnosis of IgAN before or without kidney biopsy has been a topic of discussion^6^. Specifically, the study of non-invasive diagnostic approaches for IgAN through blood and urine biomarkers has gained attention. Biomarkers such as microscopic hematuria, persistent proteinuria, serum IgA levels, and the serum IgA/C3 ratio have been identified as effective for distinguishing IgAN from other kidney diseases^7,8^. Although these variables are measurable in routine clinical settings, their diagnostic capability is limited, serving primarily to aid differential diagnosis. Recent studies have emphasized the importance of galactose-deficient IgA1 (Gd-IgA1), Gd-IgA1-specific IgG, and Gd-IgA1-containing immune complexes in IgAN pathogenesis^9,10^. Elevated serum levels of these markers have been observed in IgAN, suggesting their potential as specific biomarkers^11^. However, their practical application is limited, as their measurement requires advanced equipment not available in general medical facilities.

Machine learning, a subset of artificial intelligence, is instrumental in analyzing extensive clinical data from electronic health records, facilitating the development of predictive models^12,13^. Its application in nephrology expects advancements in predicting acute kidney injury onset^14^, prognosis of chronic kidney disease^15^, dialysis hypotension onset^16^, and assisting kidney pathological diagnosis^17^. IgAN diagnostic prediction studies have predominantly employed logistic regression^8,18,19^, a conventional statistical model assuming linear relationships and thus limiting predictive performance. Advanced machine learning algorithms, capable of modeling non-linear relationships and complex interactions, could improve predictive performance^20^. However, the efficacy of machine learning in predicting IgAN diagnosis remains unexplored.

This study aims to develop and validate diagnostic prediction models for IgAN using machine learning, based on patient demographics, blood tests, and urine tests, which can be easily obtained in clinical practice. Our other goal is to show machine learning models can be a non-invasive, highly accurate, and reliable diagnostic approach for IgAN, compared to the conventional clinical parameters or conventional statistical models.

## Methods

### Study design and study participants

**Figure 1:**
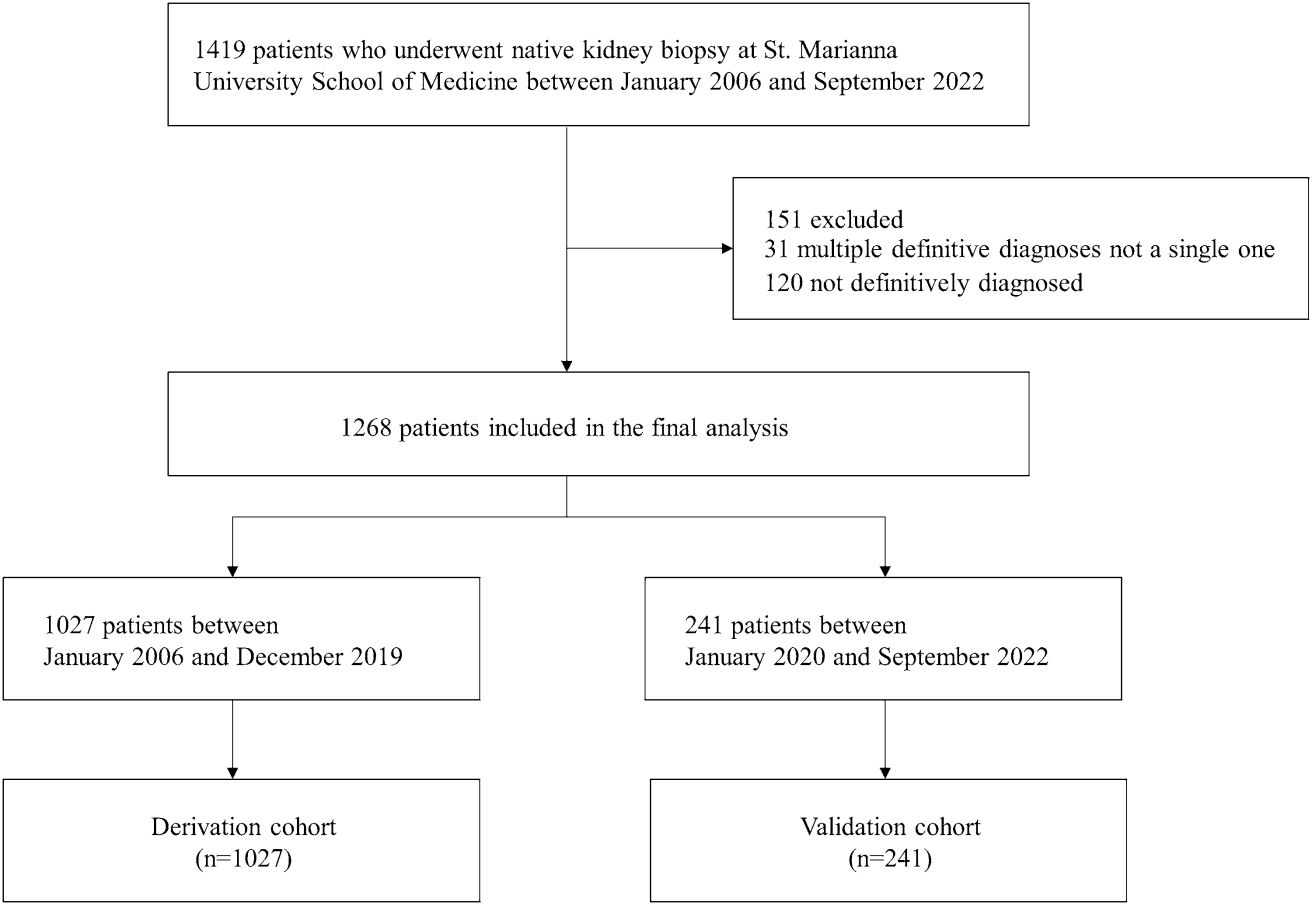
Flow diagram of patient selection.

This study is a retrospective cohort study involving patients at St. Marianna University Hospital. It included all adult patients who underwent native kidney biopsy from January 1, 2006, to September 30, 2022. Patients with inconclusive diagnoses and those with multiple primary diagnoses were excluded. The data for the cohort were collected from electronic health records, with patients who underwent kidney biopsy between January 1, 2006, and December 31, 2019, included in the derivation cohort, and those who underwent biopsy between January 1, 2020, and September 30, 2022, included in the validation cohort. Details of patient selection for the derivation and validation cohorts are shown in Fig. 1.

### Ethics approval

This study was conducted according to “The Declaration of Helsinki”, “the Transparent Reporting of a Multivariable Prediction Model for Individual Prognosis or Diagnosis Statement”^21^, and “Guidelines for Developing and Reporting Machine Learning Predictive Models in Biomedical Research: A Multidisciplinary View”^22^. The study protocol was approved by the institutional review board of St. Marianna University Hospital (approval number 6025). As the study was retrospective and involved minimal risk, the requirement for informed consent was waived.

### Predictor variables

We utilized information that is routinely measured in clinical practice as potential predictor variables. Baseline data of patients before the native kidney biopsy were retrospectively collected from electronic health records. These included demographic characteristics, blood tests, and urine tests. Demographic characteristics included age, sex, height, weight, body mass index, and blood test items comprising white blood cells, hemoglobin, total protein, albumin, blood urea nitrogen, creatinine, uric acid, aspartate aminotransferase, alanine aminotransferase, alkaline phosphatase, lactate dehydrogenase (LDH), creatine kinase (CK), total cholesterol, glucose, hemoglobin A1c, C-reactive protein, immunoglobulin G (IgG), immunoglobulin A (IgA), immunoglobulin M (IgM), complement C3, complement C4, IgA/complement C3 ratio (IgA/C3), antinuclear antibodies. Urine test items included urine protein/creatinine ratio (UPCR) and urine red blood cells (Urine RBC), with Urine RBC scored on a scale of 0 =  < 1/high power field (HPF), 2.5 = 1– 4/HPF, 7.5 = 5–9/HPF, 20 = 10–29/HPF, 40 = 30–49/HPF, 75 = 50–99/HPF, 100 =  ≥ 100/HPF.

### Outcome measures

The outcome of this study is the diagnosis of IgA nephropathy. The definitive diagnoses made through kidney biopsy by nephrologists were collected. IgA nephropathy was assigned as the correct label (1) and all other diagnoses as (0).

### Data preprocessing

The number and proportion of missing values for each variable are shown in Supplementary Table S1. Variables with more than 20% missing values were not included in the analysis. To avoid potential bias arising from excluding patients with missing data, imputation was adopted. The k-nearest neighbor imputation algorithm was employed to fill in missing values for continuous variables. The variables were standardized to have a mean of 0 and a standard deviation of 1.

### Variable selection

Effective variable selection not only enhances the learning process but also makes the results more accurate and robust. Given the importance of ensuring model reliability and trustworthiness in the medical domain, it is preferable to integrate the results of multiple feature selections instead of relying on a single feature selection^23^. Therefore, the variable reduction was performed to prevent overfitting of machine learning models and to reduce computational costs. Predictor variables with nearly zero variance, i.e., variables whose proportion of unique values was less than 5%, were excluded from the analysis. Four variable selection methods were applied to identify subsets of predictor variables. These methods included Least Absolute Shrinkage and Selection Operator, Random Forest-Recursive Feature Elimination, Random Forest-Filtering, and SelectFromModel with Extra Trees. The final predictor variables for model development were determined by integrating the results from the four methods, choosing variables that appeared three or more times across all methods.

### Model development and evaluation

For model development, the following five machine learning algorithms—eXtreme Gradient Boosting (XGBoost), LightGBM, Random Forest, Artificial Neural Network, 1 Dimentional-Convolutional Neural Network (1D-CNN)—and logistic regression were applied to the data of the derivation cohort. XGBoost and LightGBM, along with Random Forest, are tree-based algorithms that combine decision trees with ensemble learning. XGBoost and LightGBM enhance predictive performance by sequentially building decision trees and correcting the errors of previous trees by boosting techniques^24,25^. Random Forest mitigates overfitting by independently training multiple decision trees and integrating their predictions through bagging techniques^26^. Artificial Neural Networks consist of an input layer, hidden layers, and an output layer, capable of handling complex relationships between inputs and outputs using non-linear activation functions^27^. 1D-CNNs are specialized neural networks designed to process sequential data by applying convolutional filters along one dimension, effectively capturing local patterns in time-series data. This makes them particularly useful for tasks involving sequential medical data^28^. Logistic regression is a statistical linear model widely used for binary classification, producing probabilistic outputs and classifying as positive or negative based on a specific threshold^29^. To identify the optimal hyperparameters for each model, training, and validation were conducted in the derivation cohort using 5-repeated five-fold cross-validation. Bayesian optimization was used for hyperparameter tuning. The hyperparameters of each model tuned are shown in Supplementary Table S2.

The performance of the final prediction models was evaluated in both the derivation and validation cohorts. The model performance was assessed using the Area Under the Receiver Operating Characteristic curve (AUROC) and the Area Under the Precision-Recall Curve (AUPRC). The 95% confidence intervals (95% CI) for each metric were generated through 1000 bootstrap iterations with unique random seeds. AUROC and AUPRC were selected as they reflect performance across all classification thresholds and are less affected by class imbalance. The deep ROC analysis was also performed to assess discriminability in more detail^30^. Three groupings were made according to the false positive rate, and the normalized group AUROC, mean sensitivity, and mean specificity for each group were calculated. The model calibration was evaluated using calibration plots and the Brier score. Calibration plots compare the actual positive fraction to the average predicted probability across quintiles of predicted probability. The Brier score, reflecting the mean squared difference between predicted probabilities and actual outcomes, serves as a dual measure of predictive performance and calibration.

### Model interpretations

The SHapley Additive exPlanations (SHAP) method was used to explore the interpretability of the models with high diagnostic performance. SHAP provides a unified approach for the interpretation of model predictions, offering consistent and locally accurate attribution values, i.e., the SHAP values, for each variable within the predictive model^31^. The role of each variable in predicting IgA nephropathy can be explained as their collective contributions to the overall risk output for each case.

### Sensitivity analysis

Sensitivity analysis was performed to examine the differences in results caused by data split. In this analysis, ten-fold cross-validation was performed on all the data without splitting the data by the derivation cohort and the validation cohort, and adjusting hyperparameters, training, and evaluating the performance.

### Statistical analysis

Continuous variables were described using mean and standard deviation for normally distributed data, and median values along with interquartile ranges for non-normally distributed data. Categorical variables were presented as counts and percentages. For statistical comparisons, the Student's t-test was applied to normally distributed continuous variables, the Mann–Whitney U test to non-normally distributed continuous variables, and the chi-square test to categorical variables. Variables with a two-tailed p-value less than 0.05 were considered statistically significant. For variable selection, we employed the sklearn library in Python (version 3.10.12). Model development utilized the sklearn, xgboost, lightgbm, and torch libraries and evaluation was conducted using the sklearn, optuna, deeproc, and shap libraries. R (version 4.2.2) was used for statistical analyses.

### Ethics approval and consent to participate

The study was performed in accordance with the Declaration of Helsinki and Ethical Guidelines for Medical and Health Research Involving Human Subjects. The study was approved by the St. Marianna University Hospital Institutional Review Board (approval number: 6025) which allowed for analysis of patient-level data with a waiver of informed consent.

## Results

### Patient characteristics

After excluding participants with multiple primary diagnoses or without definitive diagnosis, 1268 participants were enrolled. Of these, 1027 were included in the derivation cohort and 241 in the validation cohort. The baseline characteristics and outcomes for the derivation and validation cohorts are presented in Table 1. In the derivation cohort, 294 (28.6%) were diagnosed with IgA nephropathy, compared to 59 (24.5%) in the validation cohort. The baseline characteristics of the IgA nephropathy and non-IgA nephropathy groups for each cohort are detailed in Supplementary Tables S3 and S4.

**Table 1:** Baseline characteristics and outcomes of the patients in the derivation and validation cohorts.

Variables	Derivation cohort (n = 1027)	Validation cohort (n = 241)	p-value
Demographic characteristics			
Age (years)	47 [32, 65]	59[44, 72]	< 0.001
Sex (male)	483 (47.0)	131 (54.4)	0.045
Height (cm)	161.6 [155.0, 169.0]	162.7 [155.8, 169.6]	0.301
Body Weight (kg)	58.1 [50.5, 67.4]	59.0 [50.5, 69.3]	0.361
Body Mass Index (kg/m^2^)	22.2 [19.9, 25.2]	22.3 [20.1, 25.4]	0.298
Blood tests			
White blood cells (/μL)	6500 [5200, 8300]	6100 [4500, 8100]	0.032
Hemoglobin (g/dL)	12.4 [10.6, 14.0]	12.1 [10.3, 13.5]	0.035
Total protein (g/dL)	6.5 [5.7, 7.1]	6.4 [5.7, 6.9]	0.057
Albumin (g/dL)	3.6 [2.7, 4.1]	3.3 [2.3, 3.8]	< 0.001
BUN (mg/dL)	16.5 [12.3, 23.2]	18.00 [12.8, 28.2]	0.019
Creatinine (mg/dL)	0.90 [0.69, 1.35]	1.11 [0.76, 1.67]	< 0.001
Uric acid (mg/dL)	6.1 [4.9, 7.3]	6.2 [5.0, 7.5]	0.341
AST (U/L)	20 [16, 26]	20 [17, 26]	0.412
ALT (U/L)	16 [11, 24]	16 [12, 24]	0.672
ALP (U/L)	203 [162, 259]	83 [60, 161]	< 0.001
LDH (U/L)	192 [164, 237]	204 [166, 241]	0.127
CK (U/L)	73 [44, 123]	73 [42, 111]	0.460
Total cholesterol (mg/dL)	200 [166, 243]	195 [165, 242]	0.292
Glucose (mg/dL)	98 [91, 111]	99 [92, 109]	0.407
HbA1c (%)	5.3 [4.9, 5.7]	5.6 [5.2, 5.9]	< 0.001
C-Reactive Protein (mg/dL)	0.08 [0.03, 0.52]	0.14 [0.04, 0.98]	0.002
IgG (mg/dL)	1187 [861, 1572]	1184 [817, 1547]	0.777
IgA (mg/dL)	282 [206, 383]	291 [211, 366]	0.900
IgM (mg/dL)	96 [65, 143]	82 [51, 115]	< 0.001
Complement C3 (mg/dL)	100 [82, 123]	106 [90, 132]	0.003
Complement C4 (mg/dL)	25 [18, 33]	27 [20, 35]	0.012
IgA/C3	2.83 [1.97, 4.31]	2.64 [1.85, 3.76]	0.19
ANA (titer)	40 [40, 80]	40 [40]	0.011
Urine tests			
Urine RBC (/HPF)			0.048
< 1	173 (16.8)	46 (19.1)	
1–4	191 (18.6)	50 (20.7)	
5–9	148 (14.4)	30 (12.4)	
10–29	219 (21.3)	49 (20.3)	
30–49	71 (6.9)	29 (12.0)	
50–99	71 (6.9)	14 (5.8)	
≥ 100	154 (15.0)	23 (9.5)	
UPCR (g/gCre)	1.19 [0.47, 3.74]	1.10 [0.42, 2.98]	0.143
Outcome			
IgA nephropathy	294 (28.6)	59 (24.5)	0.203

BUN, blood urea nitrogen; AST, aspartate aminotransferase; ALT, alanine aminotransferase; ALP, alkaline phosphatase; LDH, lactate dehydrogenase; CK, creatine kinase; HbA1c, hemoglobin A1c; IgG, immunoglobulin G; IgA, immunoglobulin A; IgM, immunoglobulin M; IgA/C3, immunoglobulin A/Complement C3 ratio; ANA, antinuclear antibodies; Urine RBC, urine red blood cells; UPCR, urine protein to creatinine ratio.

### Predictor variables

The predictive variables were chosen based on their appearance in at least three out of four feature selection methods applied, namely Least Absolute Shrinkage and Selection Operator, Random Forest-Recursive Feature Elimination, Random Forest-Filtering, and SelectFromModel with Extra Trees. A total of 14 variables were selected as predictors and included in the machine learning models: age, hemoglobin, total protein, albumin, LDH, CK, C-reactive protein, IgG, IgA, complement C3, complement C4, IgA/C3, Urine RBC, and UPCR (Supplementary Table S5).

### Model performance

**Figure 2:**
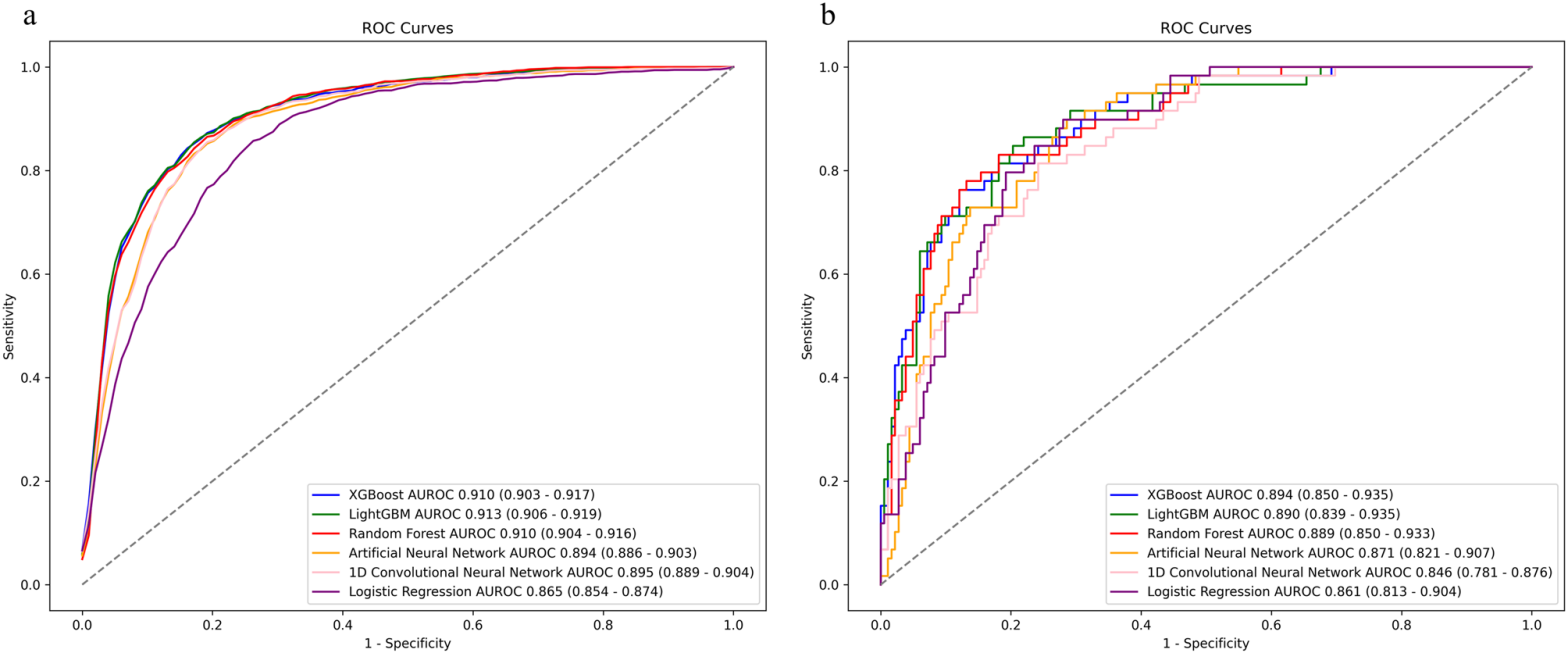
Receiver-operating characteristic curves of the machine learning models in (**a**) derivation cohort and (**b**) validation cohort.

**Figure 3:**
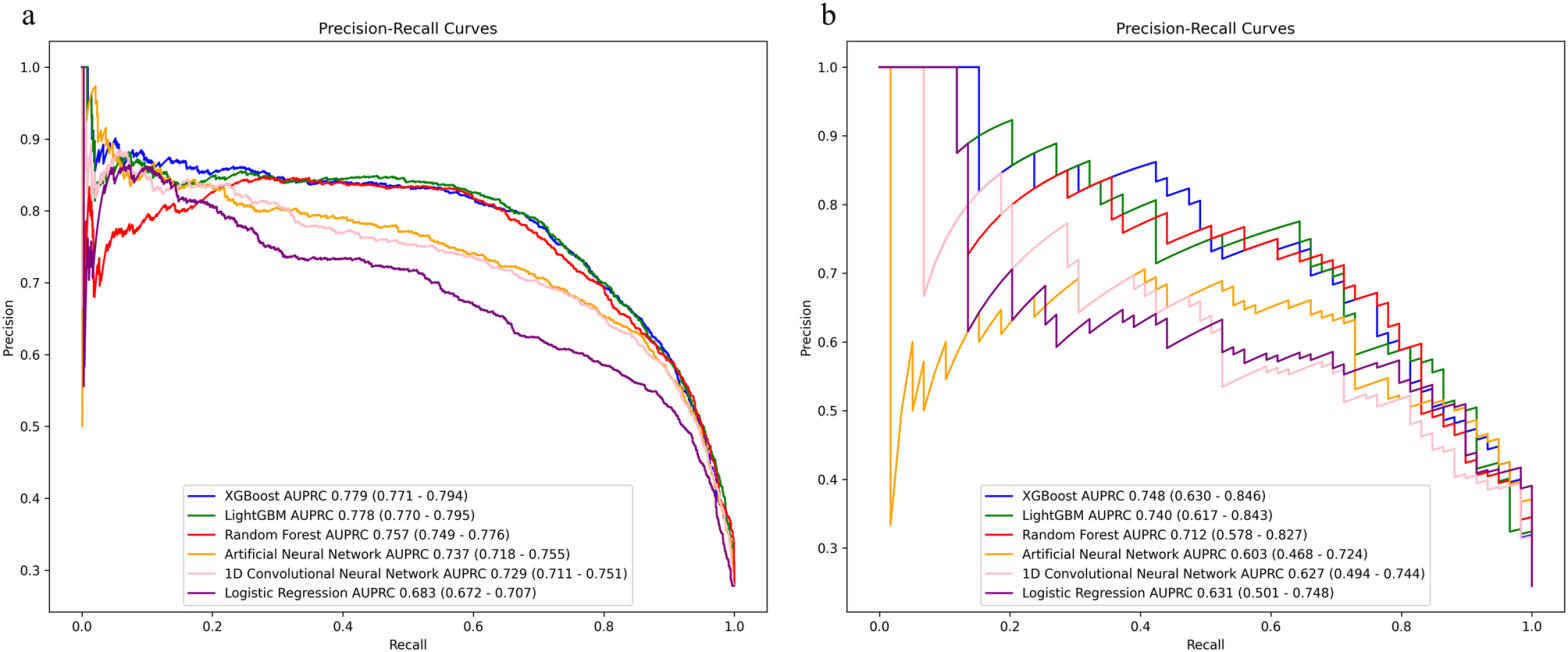
Precision-recall curves of the machine learning models in (**a**) derivation cohort and (**b**) validation cohort.

In the derivation cohort, LightGBM achieved the highest AUROC at 0.913 (95% CI 0.906–0.919), significantly higher than logistic regression, Artificial Neural Network and 1D-CNN, not significantly different from XGBoost and Random Forest (Fig. 2). In the validation cohort, XGBoost had the highest AUROC at 0.894 (95% CI 0.850–0.935), though no significant differences were observed with any models. In the derivation cohort, the AUPRC for XGBoost was 0.779 (95% CI 0.771–0.794), significantly higher than logistic regression, Artificial Neural Network and 1D-CNN with no significant difference from LightGBM and Random Forest (Fig. 3). In the validation cohort, XGBoost also scored the highest AUPRC at 0.748 (95% CI 0.630–0.846), no significant differences were found with any models. The results of the group normalized AUROC, mean sensitivity, and mean specificity for each machine learning model in the derivation and validation cohorts by the deep ROC analysis are shown in Table 2. XGBoost and LightGBM had favorable normalized group AUROC in all three groups divided by the false positive rate. The calibration plot demonstrated good calibration for all models, with the Brier Score ranging from 0.107 to 0.131 (Supplementary Fig. S1).

**Table 2:** The deep ROC analysis of the machine learning models in the derivation and validation cohort.

	Derivation cohort				Validation cohort			
FPR	[0,1]	[0.0.33]	[0.33,0.67]	[0.67,1]	[0,1]	[0.0.33]	[0.33,0.67]	[0.67,1]
Predicted probability	All	High	Medium	Low	All	High	Medium	Low
XGBoost								
AUROCni	0.910 (0.019)	0.896 (0.020)	0.914 (0.038)	0.965 (0.032)	0.894	0.871	0.905	0.965
Avg sensitivity	0.910 (0.019)	0.766 (0.043)	0.970 (0.017)	0.995 (0.006)	0.894	0.715	0.968	0.999
Avg specificity	0.910 (0.019)	0.942 (0.011)	0.582 (0.044)	0.109 (0.112)	0.894	0.928	0.592	0.308
LightGBM								
AUROCni	0.913 (0.020)	0.897 (0.020)	0.916 (0.043)	0.971 (0.032)	0.890	0.872	0.878	0.967
Avg sensitivity	0.913 (0.020)	0.770 (0.045)	0.970 (0.019)	0.996 (0.005)	0.890	0.716	0.951	1.000
Avg specificity	0.913 (0.020)	0.943 (0.014)	0.499 (0.162)	0.111 (0.119)	0.890	0.928	0.511	0.324
Random Forest								
AUROCni	0.910 (0.018)	0.894 (0.020)	0.914 (0.040)	0.971 (0.027)	0.889	0.868	0.886	1.000
Avg sensitivity	0.910 (0.018)	0.770 (0.044)	0.969 (0.018)	0.997 (0.003)	0.889	0.704	0.975	1.000
Avg specificity	0.910 (0.018)	0.942 (0.012)	0.503 (0.165)	0.150 (0.129)	0.889	0.929	0.588	0
Artificial neural network								
AUROCni	0.894 (0.017)	0.878 (0.017)	0.903 (0.038)	0.946 (0.036)	0.871	0.828	0.900	1.000
Avg sensitivity	0.894 (0.017)	0.745 (0.038)	0.954 (0.016)	0.980 (0.009)	0.871	0.638	0.973	1.000
Avg specificity	0.894 (0.017)	0.925 (0.011)	0.485 (0.158)	0.113 (0.117)	0.871	0.896	0.533	0
1D-CNN								
AUROCni	0.895 (0.042)	0.902 (0.053)	0.872 (0.047)	0.898 (0.051)	0.846	0.792	0.859	1.000
Avg sensitivity	0.895 (0.042)	0.709 (0.102)	0.918 (0.033)	0.975 (0.012)	0.846	0.500	0.950	1.000
Avg specificity	0.895 (0.042)	0.940 (0.029)	0.535 (0.036)	0.168 (0.020)	0.846	0.865	0.559	0
Logistic regression								
AUROCni	0.865 (0.022)	0.842 (0.030)	0.876 (0.029)	0.939 (0.045)	0.861	0.820	0.874	1.000
Avg sensitivity	0.865 (0.022)	0.673 (0.059)	0.953 (0.016)	0.989 (0.011)	0.861	0.614	0.967	1.000
Avg specificity	0.865 (0.022)	0.913 (0.020)	0.528 (0.058)	0.094 (0.109)	0.861	0.896	0.560	0

In the derivation cohort, the mean of the metric for each fold of the 5-repeated five-fold cross validation is calculated and the standard deviation is given in parentheses ().

FPR, false positive rate; AUROCni, group normalized area under the receiver-operating characteristic curve; Avg sensitivity, average sensitivity; Avg specificity, average specificity.

### Model interpretations

**Figure 4:**
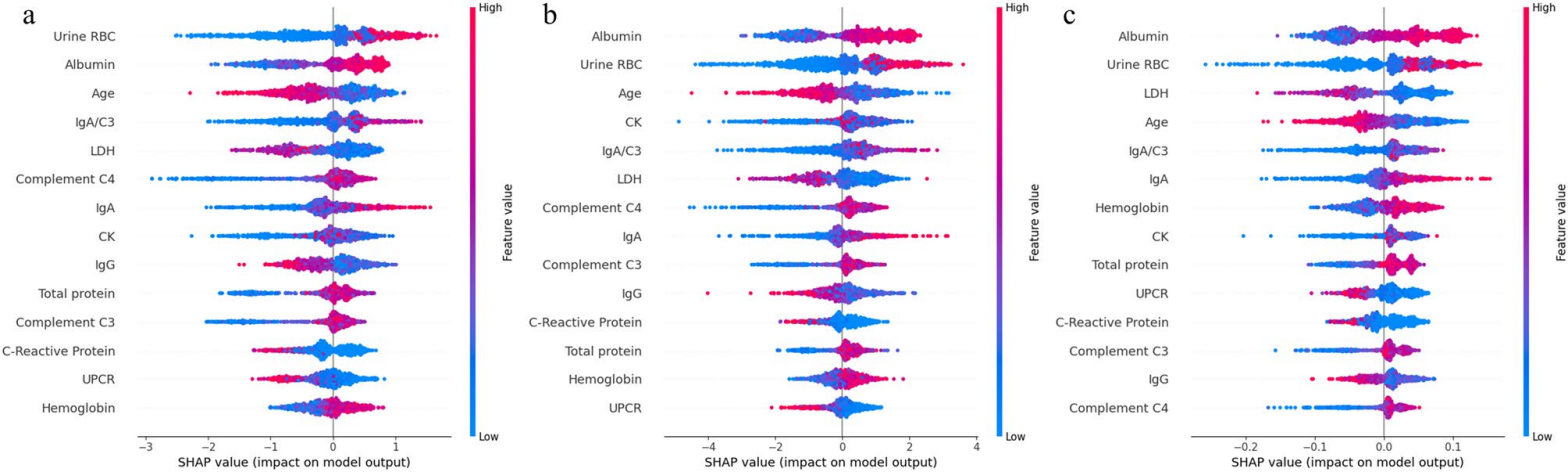
Shapley additive explanations beeswarm plots of (**a**) XGBoost, (**b**) LightGBM, and (**c**) Random Forest for prediction of IgA nephropathy. LDH, lactate dehydrogenase; CK, creatine kinase; IgG, immunoglobulin G; IgA, immunoglobulin A; IgA/C3, immunoglobulin A/Complement C3 ratio; Urine RBC, urine red blood cells; UPCR, urine protein to creatinine ratio.

SHAP values were calculated for the high-performing XGBoost, LightGBM, and Random Forest models to interpret these models. The SHAP bar plots showed the influential variables on the models' predictions (Supplementary Fig. S2). Age, albumin, IgA/C3, and Urine RBC were consistently among the top five predictor variables across all three models. Figure 4 shows the SHAP beeswarm plots, which revealed a negative correlation between age and the prediction of IgAN, while positive correlations were observed with albumin, IgA/C3, and Urine RBC. The SHAP dependence plots indicated various complex relationships between variables and the prediction of IgAN, showing similar patterns across the models (Supplementary Figs. S3–S5).

### Sensitivity analysis

The performance was evaluated using ten-fold cross-validation across the entire dataset for sensitivity analysis. LightGBM achieved the highest AUROC at 0.913 (95% CI 0.906–0.921), significantly higher than logistic regression, Artificial Neural Network and 1D-CNN, not significantly different from XGBoost and Random Forest (Supplementary Fig. S6). The AUPRC for XGBoost was 0.785 (95% CI 0.775–0.809), significantly higher than logistic regression, Artificial Neural Network and 1D-CNN with no significant difference from LightGBM and Random Forest (Supplementary Fig. S7). The calibration plot showed good calibration for all the models, with the Brier Score ranging from 0.106 to 0.129 (Supplementary Fig. S8).

## Discussion

In this study, we developed and validated machine learning-based predictive models for diagnosing IgA nephropathy. To the best of our knowledge, this is the first study to compare and evaluate the performance of multiple machine-learning models in diagnosing IgA nephropathy. We evaluated several algorithms, finding that models employing XGBoost, LightGBM, and Random Forest were effective. We confirmed key predictors like age, serum albumin, serum IgA/C3 ratio, and urine red blood cells, in line with previous findings. Additionally, it suggested that the relationships between predictive factors and IgAN predictions could extend beyond simple linearity, hinting at the importance of analyzing diverse patterns for accurate diagnosis of IgAN. These findings suggest that machine learning has potential in the non-invasive and reliable diagnosis of IgA nephropathy.

Several machine learning models were evaluated, and XGBoost, LightGBM, and Random Forest exhibited consistently superior predictive performance in the derivation and validation cohorts to the conventional logistic regression model, which we have traditionally relied on for the prediction of IgAN^8,18,19^. While logistic regression is known for its high transparency and interpretability, it assumes linear relationships between predictor variables and target variables, which can limit its performance^20,29^. Recently, the utility of clinical prediction models using machine learning has been discussed, but there are few reports on the diagnosis of IgAN^32–34^. An IgAN prediction study involving 155 patients undergoing kidney biopsy showed the utility of machine learning models, with Bayesian Networks achieving an AUROC of 0.83 and logistic regression an AUROC of 0.75^32^. Another study with 519 IgAN patients and 211 non-IgAN patients indicated the potential of Artificial Neural Networks with an AUROC of 0.839 for logistic regression and 0.881 for Artificial Neural Networks^33^. However, these studies were limited to comparisons between two models without statistical analysis using 95% confidence intervals, making it difficult to generalize the results. In a study involving 120 patients with primary glomerular diseases, the prediction model based on superb microvascular imaging-based deep learning, radiomics characteristics, and clinical factors achieved an AUROC of 0.884 (95% CI 0.773–0.996)^34^. However, the study had a small sample size, and without comparisons to other models, the robustness remains unclear. Therefore, we evaluated the performance of multiple models in a larger patient population than in previous studies. Specifically, in a cohort of 1,268 participants, six models (5 machine learning models and a conventional logistic regression model), with LightGBM performing best in the derivation phase, statistically significantly higher than Artificial Neural Network, 1D-CNN, and logistic regression, not significantly different from XGBoost and Random Forest. Previous studies evaluated various tabular data sets and showed that machine learning methods frequently outperformed logistic regression^35,36^. Recent studies have shown that tree-based machine learning models like XGBoost, LightGBM, and Random Forest outperform Neural Networks in general tabular data prediction^37,38^. The superior performance of XGBoost, LightGBM, and Random Forest in predicting IgA nephropathy is consistent with these previous findings, underscoring the potential value of tree-based machine learning models for non-invasive diagnosis of IgAN. However, no significant differences in model performance were observed in the validation phase, indicating the necessity for further verification of model generalizability.

We clarified the “black box” of XGBoost, LightGBM, and Random Forest through the SHAP method, identifying age, albumin, IgA/C3, and Urine RBC as important predictor variables. This method is a widely used explanatory technique for interpreting the contribution of predictor variables to model outputs^31,39,40^. Previous studies have reported that the presence of microscopic hematuria and/or persistent proteinuria, IgA, and IgA/C3 are useful for distinguishing IgA nephropathy from other kidney diseases^7,8^. Other research using multivariate logistic regression suggested that age, IgA/C3, albumin, IgA, IgG, eGFR, and the presence of hematuria are independent predictive variables for IgAN^33^. The key predictor variables identified in our study are in line with previous related studies. We additionally visualized the relationships between each variable and the predictions of these models through the SHAP dependence plots, discovering the possibility of various complex relationships. The findings that all three models showed similar results also suggest the importance and robustness of age, albumin, IgA/C3, and Urine RBC as predictor variables. These insights are poised to enhance our understanding of how these variables relate to IgAN moving forward.

Our findings have clinically significant implications. First, simple, accurate, and non-invasive predictive models for IgAN can be developed using similar methods, with potential for clinical application. Second, our models employ variables that are routinely collected in clinical settings, meaning that their adoption requires no additional tests or financial costs beyond standard clinical care procedures. Third, identifying key variables and visualizing their relationships with IgAN predictions could provide new perspectives for distinguishing IgAN in clinical settings.

This study has several limitations. First, it relied on the data from a single center, lacking external validation across various institutions. Assessing our model's external validity in diverse patient groups remains essential. Second, the limited sample size, particularly in the validation phase, might lead to inadequate statistical power, requiring careful interpretation of each model's evaluative performance. Third, our study cohort included all patients undergoing kidney biopsy, not focused on those with specific clinical manifestations of IgAN like chronic glomerulonephritis. This wide scope requires careful consideration before implementing our predictive models in clinical settings. Given these limitations, future studies should aim for broader validation and verification in multiple institutions to assess the generalizability and clinical potential utility of the models.

In conclusion, this study demonstrated the utility of machine learning models using common clinical data in the diagnostic prediction of IgA nephropathy. The machine learning models (XGBoost, LightGBM, and Random Forest) showed higher diagnostic performance compared to a conventional statistical model and the ability to handle complex relationships of prediction. These models can be helpful for non-invasive and reliable methods to predict IgAN.

### Supplementary Information

Supplementary Information.

## Data Availability

The dataset cannot be disclosed as approval has not been received from the Ethics Committee of St Marianna University Hospital. The code underlying this article will be shared on reasonable request to the corresponding author.
