# Supplementary material for: Machine learning-based diagnostic prediction of IgA nephropathy: model development and validation study
Source: Sci Rep. 2024 May 30;14:12426. doi: 10.1038/s41598-024-63339-7 (PMC11139869; doi:10.1038/s41598-024-63339-7)

Supplementary Table S1. Data types and missing values for the variables of interest.

Variables	Data types	Missing Values, n (%)	
		Derivation cohort	Validation cohort
Age	Continuous	0 (0.0)	0 (0.0)
Sex	Categorical	0 (0.0)	0 (0.0)
Height	Continuous	12 (1.17)	5 (2.07)
Body Weight	Continuous	3 (0.29)	0 (0.0)
Body Mass Index	Continuous	14 (1.36)	5 (2.07)
White blood cells	Continuous	2 (0.19)	1 (0.41)
Hemoglobin	Continuous	2 (0.19)	1 (0.41)
Total protein	Continuous	5 (0.49)	2 (0.83)
Albumin	Continuous	34 (3.31)	0 (0.0)
BUN	Continuous	3 (0.29)	0 (0.0)
Creatinine	Continuous	3 (0.29)	0 (0.0)
Uric acid	Continuous	34 (3.31)	3 (1.24)
AST	Continuous	2 (0.19)	0 (0.0)
ALT	Continuous	3 (0.29)	0 (0.0)
ALP	Continuous	51 (4.97)	9 (3.73)
LDH	Continuous	7 (0.68)	0 (0.0)
CK	Continuous	61 (5.94)	8 (3.32)
Total cholesterol	Continuous	108 (10.52)	12 (4.98)
Glucose	Continuous	99 (9.64)	12 (4.98)
HbA1c	Continuous	187 (18.21)	36 (14.94)
C-Reactive Protein	Continuous	24 (2.34)	1 (0.41)
IgG	Continuous	78 (7.59)	22 (9.13)
IgA	Continuous	89 (8.67)	24 (9.96)
IgM	Continuous	108 (10.52)	28 (11.62)
Complement C3	Continuous	107 (10.42)	25 (10.37)
Complement C4	Continuous	108 (10.52)	28 (11.62)
IgA/C3	Continuous	133 (12.95)	33 (13.69)
ANA	Continuous	124 (12.07)	35 (14.52)
Urine RBC	Categorical	0 (0.0)	0 (0.0)
UPCR	Continuous	123 (11.98)	0 (0.0)

BUN: Blood Urea Nitrogen, AST: Aspartate Aminotransferase, ALT: Alanine Aminotransferase, ALP: Alkaline Phosphatase, LDH: Lactate Dehydrogenase, CK: Creatine Kinase, HbA1c: Hemoglobin A1c, IgG: Immunoglobulin G, IgA: Immunoglobulin A, IgM: Immunoglobulin M,

IgA/C3: Immunoglobulin A / Complement C3 ratio, ANA: Antinuclear antibodies , Urine RBC: Urine red blood cells, UPCR: Urine protein to creatinine ratio.

Supplementary Table S2. Tuning parameters used for each machine learning algorithm.

Algorithm	Packages	Tuning Parameters
XGBoost	xgboost	lambda, alpha, colsample_bytree, subsample, learning_rate, n_estimators, max_depth, min_child_weight
LightGBM	lightgbm	lambda_l1, lambda_l2, num_leaves, feature_fraction, bagging_fraction, bagging_freq, min_child_samples
Random Forest	sklearn.ensemble	n_estimators, max_depth, min_samples_split, min_samples_leaf, max_features
Logistic Regression	sklearn.linear_model	C
Artificial Neural Network	sklearn.neural_network	hidden_layer_sizes, activation, alpha, learning_rate, max_iter
1D Convolutional Neural Network	torch	learning_rate, num_filters, kernel_size, num_epochs, batch_size

Supplementary Table S3. Baseline characteristics of patients with and without IgA nephropathy in the derivation cohort.

Variables	non-IgAN (n=733)	IgAN (n=294)	p-value
**Demographic characteristics**			
Age (years)	53 [36, 68]	37 [26, 52]	<0.001
Sex (male)	322 (43.9)	161 (54.8)	0.002
Height (cm)	160.5 [153.9, 168.0]	164.3 [157.8, 171.0]	<0.001
Body Weight (kg)	58.0 [49.6, 66.8]	58.7 [51.8, 68.7]	0.056
Body Mass Index (kg/m^2)	22.4 [20.0, 25.4]	21.9 [19.8, 24.4]	0.188
**Blood tests**			
White blood cells (/μL)	6600 [5200, 8700]	6200 [5225, 7575]	0.056
Hemoglobin (g/dL)	11.9 [10.2, 13.5]	13.4 [12.1, 14.5]	<0.001
Total protein (g/dL)	6.3 [5.2, 7.0]	6.9 [6.4, 7.3]	<0.001
Albumin (g/dL)	3.2 [2.5, 3.8]	4.1 [3.7, 4.3]	<0.001
BUN (mg/dL)	17.2 [12.9, 25.3]	14.9 [11.8, 19.1]	<0.001
Creatinine (mg/dL)	0.91 [0.69, 1.48]	0.89 [0.71, 1.17]	0.136
Uric acid (mg/dL)	6.1 [4.8, 7.3]	6.1 [5.0, 7.4]	0.277
AST (U/L)	21 [16, 27]	18 [16, 22]	<0.001
ALT (U/L)	16 [12, 25]	14 [11, 20]	0.002
ALP (U/L)	210 [164, 262]	195 [155, 234]	0.001
LDH (U/L)	205 [171.75, 254.25]	173 [153, 192]	<0.001
CK (U/L)	67 [36, 126]	82 [61, 115]	<0.001
Total cholesterol (mg/dL)	207 [168, 264]	188 [161, 216]	<0.001
Glucose (mg/dL)	99 [91, 113]	97 [91, 104]	0.002
HbA1c (%)	5.4 [5.0, 5.8]	5.1 [4.9, 5.5]	<0.001
C-Reactive Protein (mg/dL)	0.13 [0.03, 0.78]	0.04 [0.03, 0.14]	<0.001
IgG (mg/dL)	1227.5 [775, 1664]	1132 [941, 1385]	0.199
IgA (mg/dL)	258 [183, 359]	318 [247, 424]	<0.001
IgM (mg/dL)	96[62, 144]	97 [70, 137]	0.445
Complement C3 (mg/dL)	101 [72, 126]	99 [92, 116]	0.398
Complement C4 (mg/dL)	24 [14, 34]	25 [21, 31]	0.142
IgA/C3	2.52 [1.73, 4.37]	3.10 [2.54, 4.16]	<0.001
ANA (titer)	40 [40, 160]	40 [40, 40]	<0.001
**Urine tests**			
Urine RBC (/HPF)			<0.001
< 1	160 (21.8)	13 (4.4)	

1~4	162 (22.1)	29 (9.9)	
5~9	107 (14.6)	41 (14.0)	
10~29	135 (18.4)	84 (28.6)	
30~49	44 (6.0)	27 (9.2)	
50~99	41 (5.6)	30 (10.2)	
≥ 100	84 (11.5)	70 (23.8)	
UPCR (g/gCre)	1.70 [0.59, 4.90]	0.71 [0.30, 1.43]	<0.001

BUN: Blood Urea Nitrogen, AST: Aspartate Aminotransferase, ALT: Alanine Aminotransferase, ALP: Alkaline Phosphatase, LDH: Lactate Dehydrogenase, CK: Creatine Kinase, HbA1c: Hemoglobin A1c, IgG: Immunoglobulin G, IgA: Immunoglobulin A, IgM: Immunoglobulin M, IgA/C3: Immunoglobulin A / Complement C3 ratio, ANA: Antinuclear antibodies , Urine RBC: Urine red blood cells, UPCR: Urine protein to creatinine ratio.

Supplementary Table S4. Baseline characteristics of patients with and without IgA nephropathy in the validation cohort.

Variables	non-IgAN (n=182)	IgAN (n=59)	p-value
**Demographic characteristics**			
Age (years)	64 [47, 74]	49 [39, 58]	<0.001
Sex (male)	104 (57.1)	27 (45.8)	0.135
Height (cm)	163 [156, 169]	161 [156, 171]	0.911
Body Weight (kg)	58.9 [50.6, 69.5]	60.3 [50.5, 67.1]	0.946
Body Mass Index (kg/m^2)	22.6 [20.1, 25.5]	21.8 [20.2, 25.1]	0.782
**Blood tests**			
White blood cells (/μL)	6200 [4400, 8400]	6100 [4700, 7150]	0.398
Hemoglobin (g/dL)	11.7 [9.7, 13.2]	13.3 [12.1, 14.0]	<0.001
Total protein (g/dL)	6.2 [5.5, 6.8]	6.7 [6.4, 7.1]	<0.001
Albumin (g/dL)	3.0 [2.1, 3.6]	3.7 [3.6, 4.1]	<0.001
BUN (mg/dL)	19.4 [14.1, 33.8]	14.2 [11.5, 18.1]	<0.001
Creatinine (mg/dL)	1.18 [0.81, 1.88]	0.94 [0.69, 1.18]	0.001
Uric acid (mg/dL)	6.1 [4.9, 7.6]	6.5 [5.5, 7.2]	0.395
AST (U/L)	21 [17, 27]	19 [17, 23]	0.089
ALT (U/L)	16 [12, 25]	15 [12, 21]	0.46
ALP (U/L)	82 [62, 165]	80 [57, 138]	0.159
LDH (U/L)	217 [172, 250]	175 [157, 199]	<0.001
CK (U/L)	66 [37, 113]	87 [62, 108]	0.02
Total cholesterol (mg/dL)	192 [161, 253]	201 [174, 223]	0.937
Glucose (mg/dL)	99 [91, 112]	99 [94, 103]	0.487
HbA1c (%)	5.7 [5.3, 6.1]	5.4 [5.1, 5.5]	<0.001
C-Reactive Protein (mg/dL)	0.27 [0.05, 1.41]	0.04 [0.03, 0.14]	<0.001
IgG (mg/dL)	1202 [766, 1603]	1103 [962, 1352]	0.38
IgA (mg/dL)	286 [208, 364]	316 [232, 373]	0.147
IgM (mg/dL)	78 [50, 109]	93 [62, 137]	0.02
Complement C3 (mg/dL)	112 [89, 136]	97 [90, 109]	0.017
Complement C4 (mg/dL)	29 [20, 37]	24 [19, 30]	0.045
IgA/C3	2.49 [1.77, 3.72]	3.22 [2.49, 4.03]	0.026
ANA (titer)	40 [40, 40]	40 [40, 40]	0.037
**Urine tests**			
Urine RBC (/HPF)			<0.001
< 1	42 (23.1)	4 (6.8)	

1~4	41 (22.5)	9 (15.3)	
5~9	22 (12.1)	8 (13.6)	
10~29	33 (18.1)	16 (27.1)	
30~49	21 (11.5)	8 (13.6)	
50~99	9 (4.9)	5 (8.5)	
≥ 100	14 (7.7)	9 (15.3)	
UPCR (g/gCre)	1.32 [0.46, 3.61]	0.67 [0.37, 1.42]	0.002

BUN: Blood Urea Nitrogen, AST: Aspartate Aminotransferase, ALT: Alanine Aminotransferase, ALP: Alkaline Phosphatase, LDH: Lactate Dehydrogenase, CK: Creatine Kinase, HbA1c: Hemoglobin A1c, IgG: Immunoglobulin G, IgA: Immunoglobulin A, IgM: Immunoglobulin M, IgA/C3: Immunoglobulin A / Complement C3 ratio, ANA: Antinuclear antibodies , Urine RBC: Urine red blood cells, UPCR: Urine protein to creatinine ratio.

Supplementary Table S5. Predictor variables selected by the 4 variable selection methods.

Variables	Least Absolute Shrinkage and Selection Operator	Recursive feature elimination	Filtering	SelectFromModel with ExtraTrees Classifier
Age	✓	✓	✓	✓
Hemoglobin	✓	✓	✓	✓
Total protein		✓	✓	✓
Albumin	✓	✓	✓	✓
LDH	✓	✓	✓	✓
CK	✓	✓	✓	
C-Reactive Protein	✓	✓	✓	
IgG	✓	✓	✓	
IgA	✓	✓	✓	✓
Complement C3	✓	✓	✓	✓
Complement C4	✓	✓	✓	✓
IgA/C3	✓	✓	✓	✓
Urine RBC	✓	✓	✓	✓
UPCR	✓	✓	✓	✓

Supplementary Figure S1. Calibration plots of the machine learning models for IgA nephropathy prediction in (a) the derivation cohort and (b) the validation cohort.

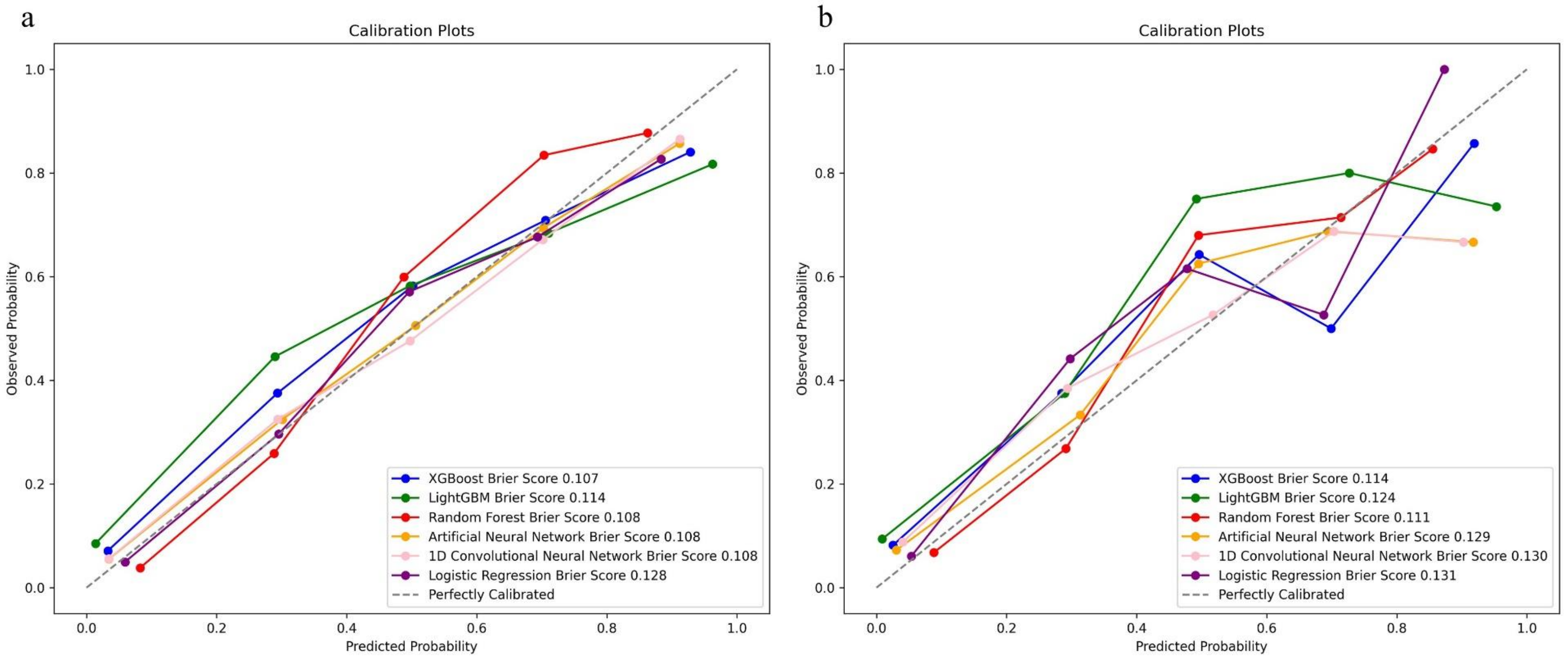

Supplementary Figure S2. Shapley additive explanations bar plots of (a) XGBoost, (b) LightGBM, and (c) Random Forest for prediction of IgA nephropathy. LDH: Lactate Dehydrogenase, CK: Creatine Kinase, HbA1c: Hemoglobin A1c, IgG: Immunoglobulin G, IgA: Immunoglobulin A, IgM: Immunoglobulin M, IgA/C3: Immunoglobulin A / Complement C3 ratio, Urine RBC: Urine Red Blood Cells Counts, UPCR: Urine protein to creatinine ratio.

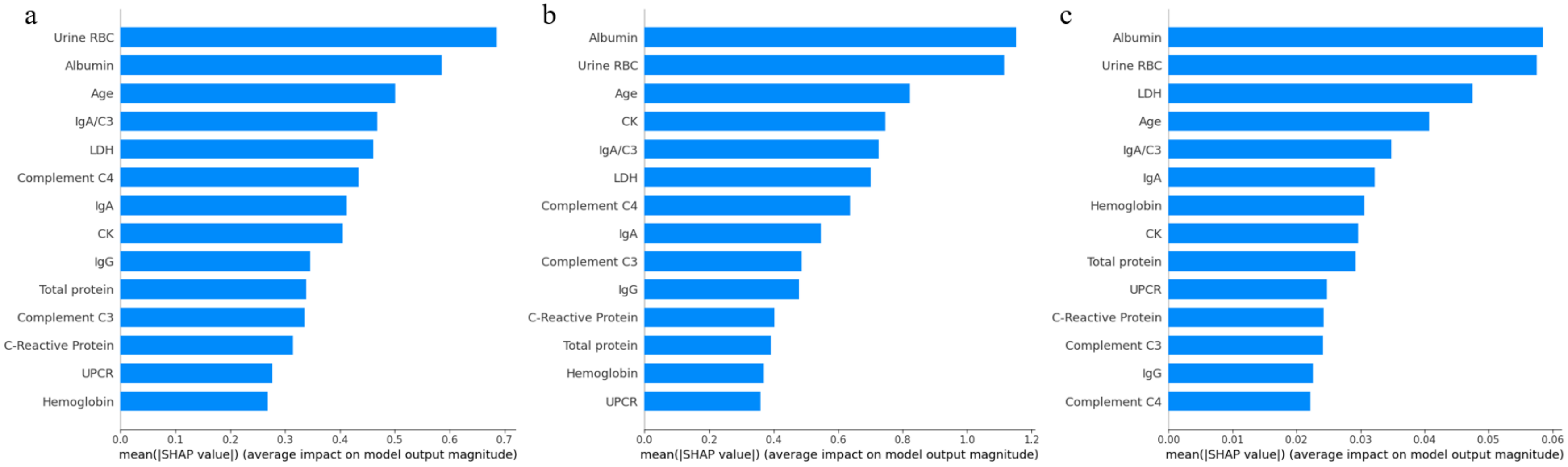

Supplementary Figure S3. Shapley additive explanations dependence plots for the association between the predictive variables and IgA nephropathy in XGBoost.

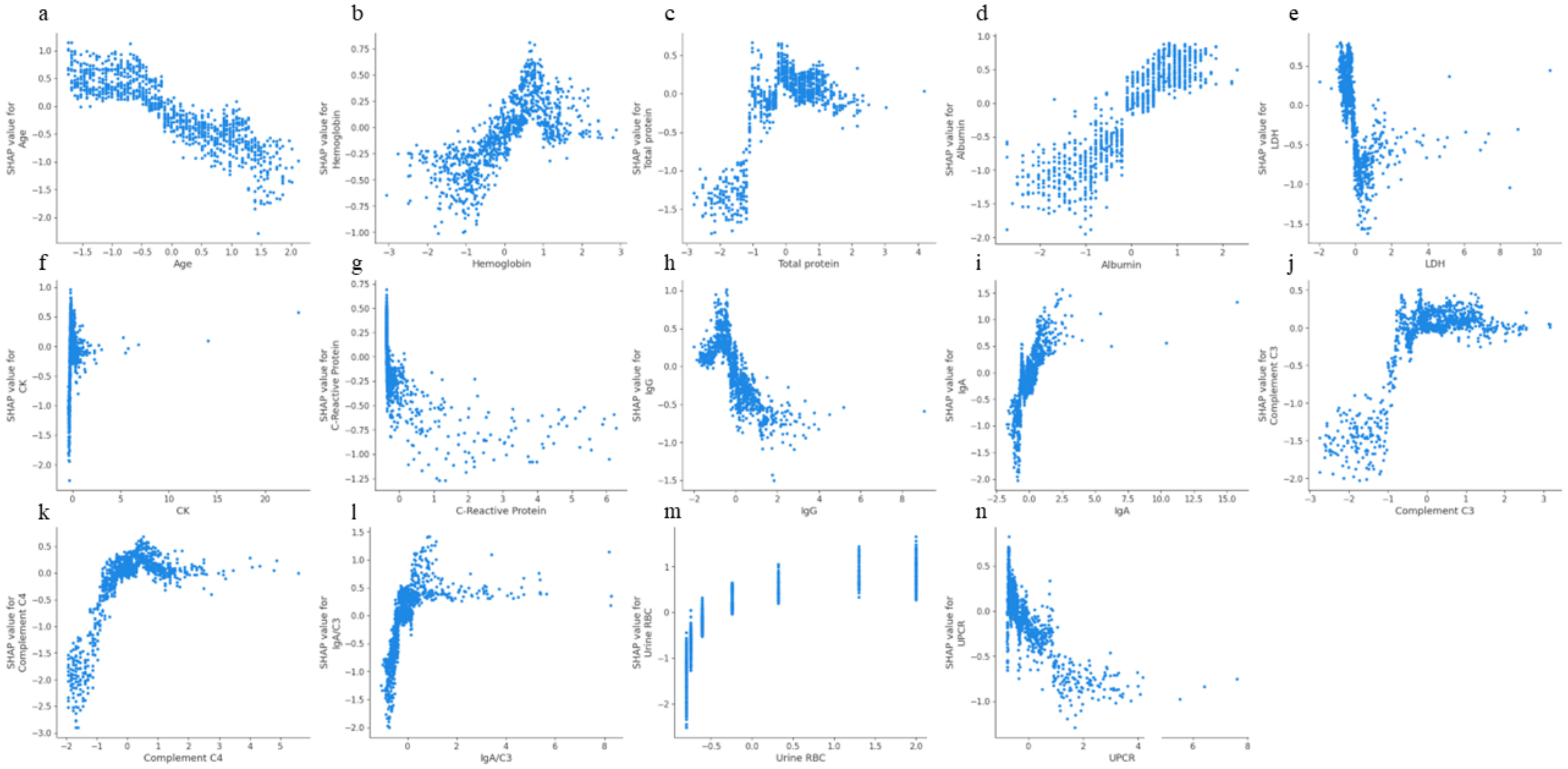

Supplementary Figure S4. Shapley additive explanations dependence plots for the association between the predictive variables and IgA nephropathy in LightGBM.

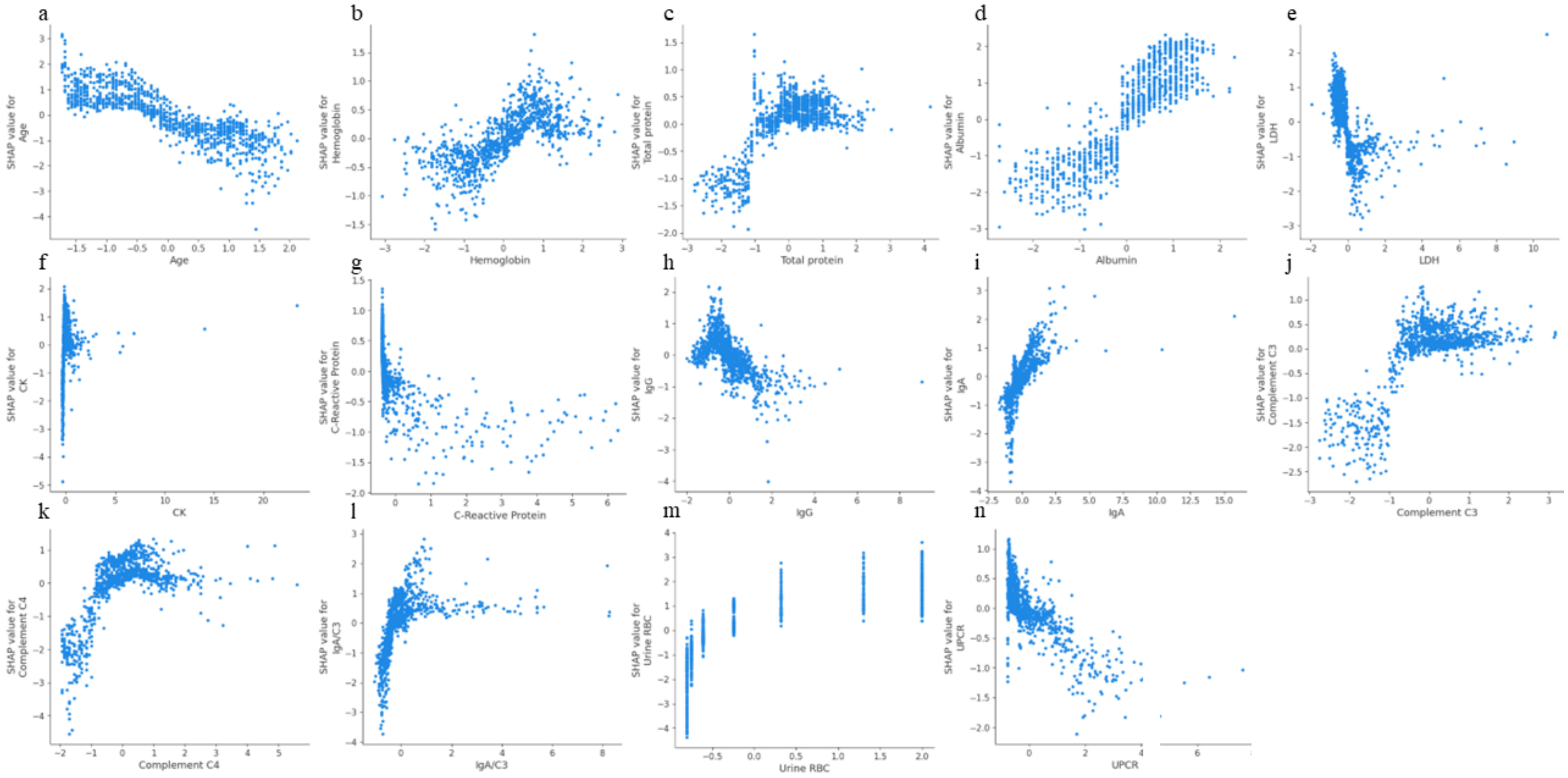

Supplementary Figure S5. Shapley additive explanations dependence plots for the association between the predictive variables and IgA nephropathy in Random Forest.

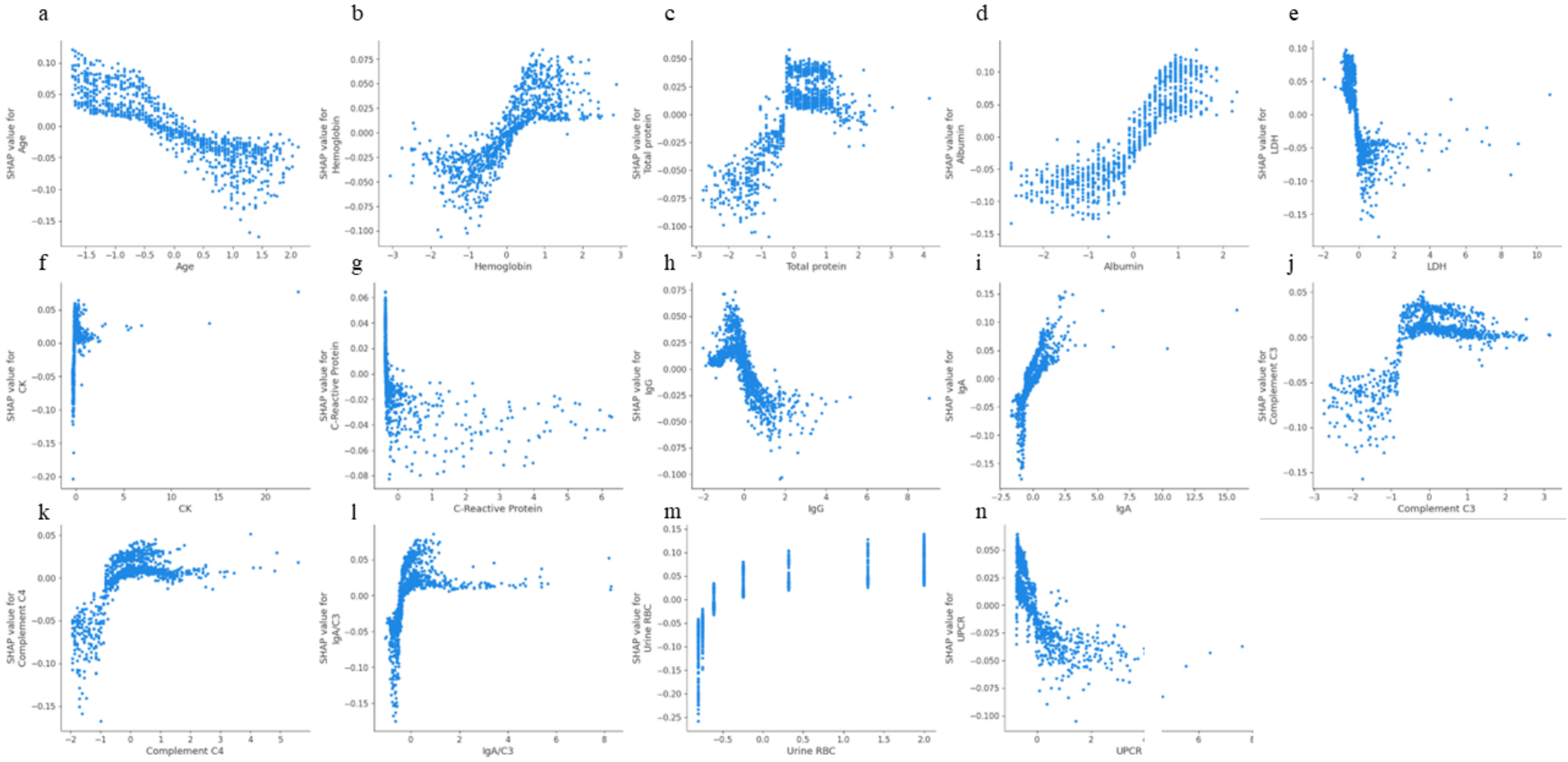

Supplementary Figure S6. Receiver-operating characteristic curves of the machine learning models using 10-fold cross-validation on the entire dataset.

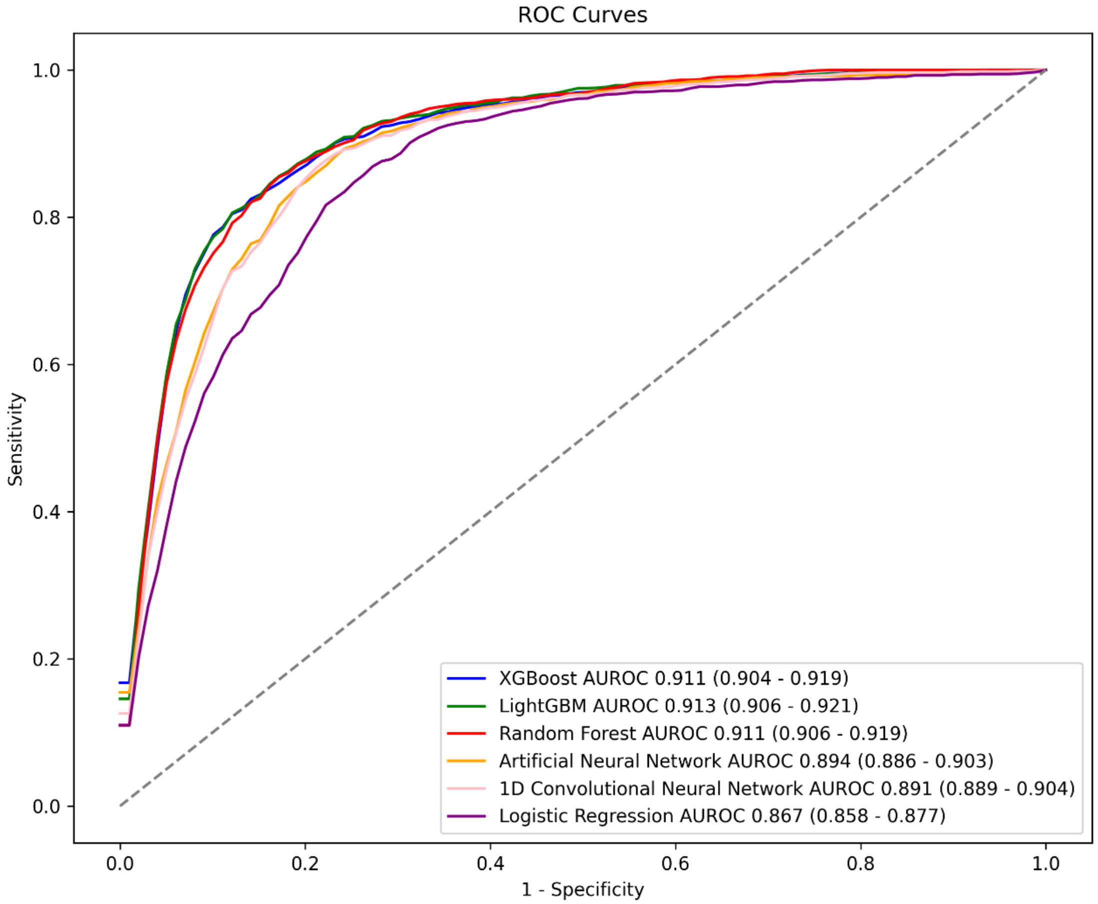

Supplementary Figure S7. Precision-recall curves of the machine learning models using 10-fold cross-validation on the entire dataset.

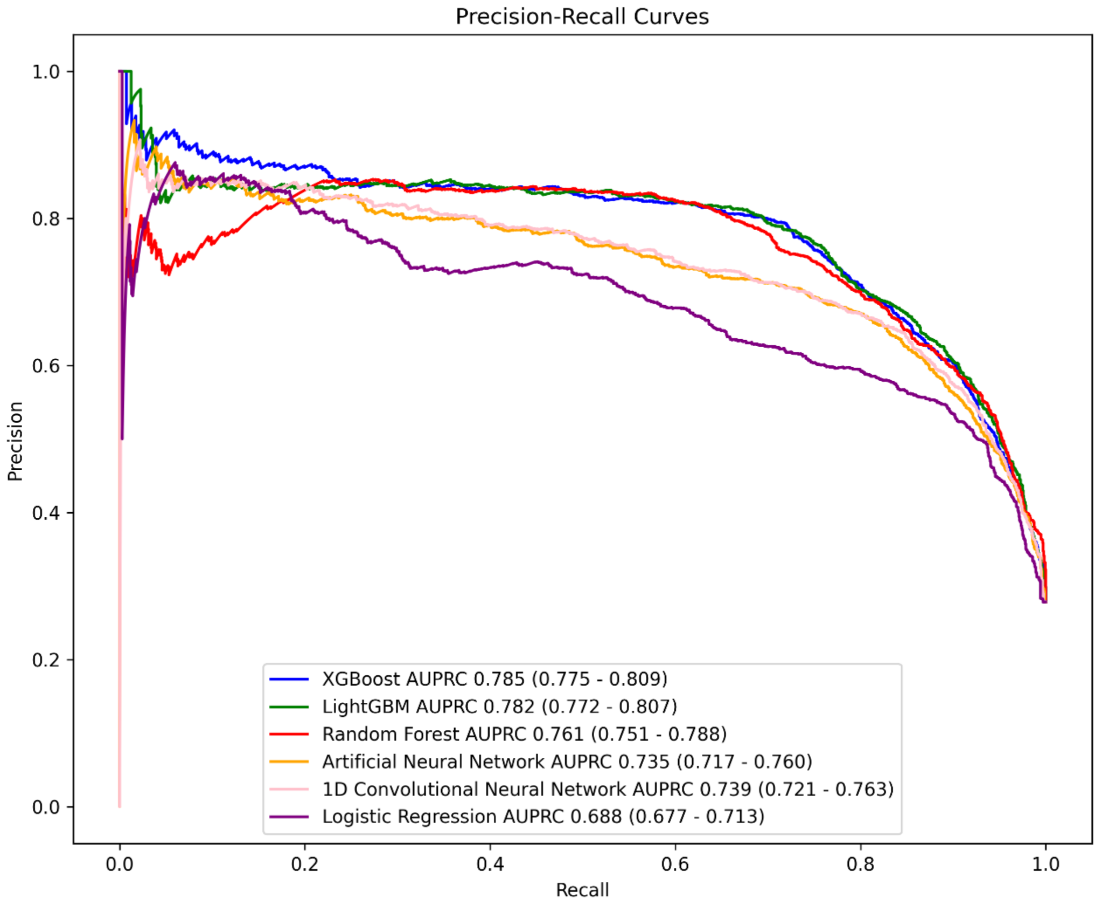

Supplementary Figure S8. Calibration plots of the machine learning models using 10-fold cross-validation on the entire dataset.

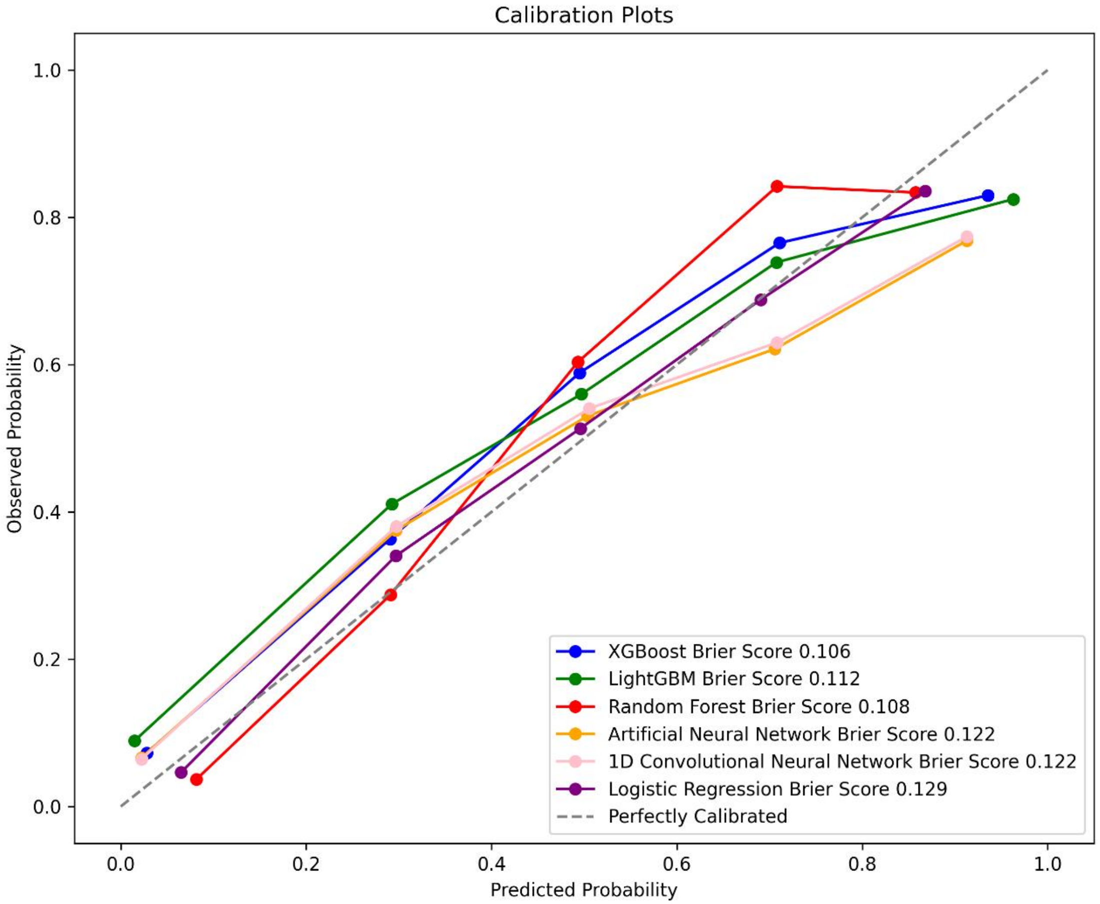

Supplement: Supplementary file 1 — Supplementary Information. [file 41598_2024_63339_MOESM1_ESM.pdf]
